# Changes of membrane fatty acids and proteins of *Shewanella putrefaciens* treated with cinnamon oil and gamma irradiation

**DOI:** 10.1186/s40643-017-0140-1

**Published:** 2017-01-30

**Authors:** Fei Lyu, Fei Gao, Qianqian Wei, Lin Liu

**Affiliations:** Department of Food Science, Ocean College, Zhejiang University of Technology, 18 Chaowang Road, Hangzhou, 310014 China

**Keywords:** Gamma irradiation, *Shewanella putrefaciens*, Cinnamon oil, Fatty acids

## Abstract

**Background:**

In order to detect the antimicrobial mechanism of combined treatment of cinnamon oil and gamma irradiation (GI), the membrane fatty acids and proteins characteristics of *Shewanella putrefaciens* (*S. putrefaciens*) treated with cinnamon oil and GI, and the distribution of cinnamon oil in *S. putrefaciens* were observed in this study.

**Results:**

The membrane lipid profile of *S. putrefaciens* was notably damaged by treatments of cinnamon oil and the combination of cinnamon oil and GI, with significantly fatty acids decrease in C14:0, C16:0, C16:1, C17:1, C18:1 (*p* < 0.05). The SDS-PAGE result showed that GI did not have obvious effect on membrane proteins (MP), but GI combined with cinnamon oil changed the MP subunits. Cinnamaldehyde, the main component of cinnamon oil, can not transport into *S. putrefaciens* obviously. It was transformed into cinnamyl alcohol in the nutrient broth with the action of *S. putrefaciens*. This indicated that the antimicrobial action of cinnamon oil mainly happened on the membrane of *S. putrefaciens.*

**Conclusion:**

Cinnamon oil could act on the membrane of *S. putrefaciens* with the damage of fatty acids and proteins, and GI would increase the destructive capability of cinnamon oil on the membrane fatty acids and proteins of *S. putrefaciens*.

## Background

The interest of the food industry and consumers in natural antimicrobials to prevent spoilage and pathogenic microorganisms has increased significantly. *Shewanella putrefaciens* has been proved to be one of the main specific spoilage organisms (SSO) presented in refrigerated meat and fish products (Borch et al. 1996; Gram and Huss 1996; Gram et al. 1990; Leblanc et al. 2001). It can generate hydrogen sulfide (H_2_S) from cysteine, reduce trimethylamine oxide (TMAO) to trimethylamine (TMA), participate in the proteolytic and lipolytic degradations, thus producing unpleasant off-odors leading to organoleptic alterations of food products (Gennari et al. 1999; Leblanc et al. 2001; López-Caballero et al. 2001; Stenström and Molin 1990). Nowadays, many studies have focused on the antimicrobial techniques against *S. putrefaciens* in different foods (Cai et al. 2015; Jasour et al. 2015; Shokri et al. 2015; Zhang et al. 2015b).

Essential oils (EOs) are characterized by a wide range of volatile compounds, some of which are important to food flavor quality, and they are generally recognized as safe (GRAS) (Belletti et al. 2004). Cinnamon oil has a strong antimicrobial activity against Gram-positive and Gram-negative bacteria (Almariri and Safi 2014; Urbaniak et al. 2014). It has been proved that cinnamon oil used in fish and meat products could extend their microbial shelf life (Van Haute et al. 2016). Cinnamaldehyde, the main component of cinnamon oil, has been shown to be effective against a broad spectrum of food-borne pathogens (Burt 2004; Holley and Patel 2005). It is common for reviewers of spice oils to ascribe the interactions of spice oils with the cell membrane (Brul and Coote 1999; Roller and Board 2003). Gill and Holley (2004) observed that there was a rapid decline in cellular adenosine triphosphate (ATP) in *Listeria monocytogenes* treated with cinnamaldehyde. It was hypothesized that cinnamaldehyde acted as an ion transporter and interacted with the cell membrane causes disruption sufficient to disperse the proton motive force by leakage of small ions and inhibition of energy generation (Gill and Holley 2004). Hammer and Heel (2012) demonstrated that cinnamaldehyde could decrease the membrane polarity before increasing the membrane permeability. It was also reported that cell membrane integrity of *Escherichia coli* and *Staphylococcus aureus* was damaged by cinnamaldehyde (Shen et al. 2015). Mousavi et al. (2016) successfully demonstrated that cinnamaldehyde could change *E. coli* metabolism through interactions with different biochemical families such as proteins, nucleic acids, lipids, and carbohydrates.

Irradiation technology has been used for decontamination and/or sterilization of dehydrated vegetables, fruits, meats, poultry, fish, and seafood in order to improve product safety and shelf life (Arvanitoyannis et al. 2009; Lacroix and Ouattara 2000). The action of gamma irradiation (GI) on DNA molecules and cell division inhibition is now well understood (Bonura et al. 1975; Le-Tien et al. 2007). Various reactive oxygen species (ROS) are produced during the irradiation treatment of foodstuff which contributes to cellular damage (Bonura et al. 1975). Although much literature has reported the mechanism of cinnamon oil and GI on bacteria alone against different bacteria, the combined antimicrobial mechanism of cinnamon oil and GI on *S. putrefaciens* has not been reported. The aim of the experiments was to evaluate the membrane damage capacity of the combination treatments of cinnamon oil and GI on *S. putrefaciens* by analyzing the membrane protein and fatty acid profiles as well as the distribution of cinnamaldehyde in *S. putrefaciens,* thus to analyze the antimicrobial mechanism of the combination treatment against *S. putrefaciens.*

## Methods

### Antimicrobial compounds

Cinnamon oil was extracted from *Cinnamomum zeylanicum* leaves by steam distillation method. It was purchased from Erin Limited Company, Australia. Cinnamon oil stock solution was prepared by emulsifying cinnamon oil in deionized water with 1% Tween-80 by stirring 30 min to get a colloidal suspension for use within 24 h with final cinnamon oil concentrations of 207 and 414 mg/mL, respectively.

### Chemicals and reagents

Cinnamaldehyde [99.5%, chromatographic pure (GCP)] and cinnamyl alcohol (99%, GCP) were purchased from Aladdin, Shanghai, China. HPLC-grade methylene dichloride was purchased from Tianjin Shield Specialty Chemical Co., Ltd., Tianjin, China. HPLC-grade acetonitrile and methanol were purchased from Tedia Company, Inc., Ohio, USA. Other solvents and chemicals were purchased from Dingguo biological technology Co., Ltd., Shanghai, China. Ultrapure water was purified on a Milli-Q system (Millipore, Bedford, USA). Millipore syringe filters (Millex-GP, 0.22 mm pore size) were purchased from Nihon Millipore, Tokyo, Japan.

### *Shewanella putrefaciens* preparation

*Shewanella putrefaciens* was isolated from spoiled fish and identified by China Center of Industrial Culture Collection. When shipped to our laboratory, the strain was cultured twice in nutrient broth (NB) at 30 °C for 24 h, then streaked on nutrient agar (NA) slants and cultured under the same conditions. The slants were stored at 4 °C and sub-cultured monthly until use. Before each experiment, stock cultures were propagated through two consecutive 24-h growth cycles in NB at 30 °C and then cultivated to the exponential phase (5 h). The working cultures contained approximately 10^8^ CFU/mL *S. putrefaciens* were obtained by diluting the exponential phase cells in nutrient broth.

### Treatments of *S. putrefaciens*

Each 50 mL working culture containing approximately 10^8^ colony-forming unit (CFU)/mL *S. putrefaciens* was transferred into 100-mL test tube. These test tubes were treated as follows: group one without adding cinnamon oil was used as control (CK); group two was added cinnamon oil with the final concentration of 207 μg/mL (C1); group three contained 207 μg/mL cinnamon oil and irradiated by GI (C1+G); group four only was irradiated (G); group five was added cinnamon oil with the final concentration of 414 μg/mL (C2). G and C1+G were irradiated 0.080 kGy GI as soon as possible after cinnamon oil treatment at the Institute of Crops and Nuclear Technology Utilization, Zhejiang Academy of Agricultural Sciences, China. The data of 207 and 414 μg/mL were the ½ MIC (minimal inhibitory concentration) and MIC values of cinnamon oil for 10^8^ CFU/mL *S. putrefaciens*. The doses were conducted by using 55-cm distance to the irradiation source with 30 min. The self-contained GI source was ^137^Cs with an approximate dose rate of 0.10 kGy/min. The dose rate was established by using National Physical Laboratory (Middlesex, United Kingdom) dosimeters. After irradiation, all samples were transported to our laboratory within 180 min. Moreover, the nutrient broths containing 207 and 414 μg/mL cinnamon oil without *S. putrefaciens* were also used as controls marked as CC1 and CC2, respectively. Each treatment group contained 5 test tubes. The assays were tested in triplicate, and values are presented as mean ± standard deviation of replicated measurements.

### Cinnamon oil analysis

The cinnamon oil was analyzed using a GC–MS system (Agilent 7890A/5975C) on a HP-5MS capillary column (30.0 m × 250 μm × 0.25 μm) using helium as the carrier gas with a split flow was 1.5 mL/min and a 100:1 split ratio. The initial oven temperature of GC was 50 °C, and programmed to 250 °C at a rate of 30 °C/min and then kept constant at 250 °C for 10 min. The inject volume was 0.5 μL and the source temperature was 230 °C. MS was taken at 70 eV and a mass range of 29–450 amu. A library search was carried out using NIST98.L database. Relative percentage amount was calculated from total ions chromatograms (TIC) by the computer (Ooi et al. 2006).

### Analysis of membrane fatty acids in *S. putrefaciens*

The working cultures of *S. putrefaciens* with different treatments at 180 min were centrifuged for 10 min at 5000*g*, and the cell pellet was harvested and re-suspended in phosphate-buffered saline (0.1 M, pH 7.0), afterwards frozen at −80 °C and freeze-dried using a freeze-dryer (FD-1-50, Bo Yikang Co. Ltd., Beijing, China). The samples were submitted for membrane fatty acid extraction. Extraction of fatty acid from cellular materials was carried out as described by Evans et al. (1998).

Lipid samples were trans-methylated for analysis of their acyl groups as fatty acid methyl esters (FAME). The samples of total lipid extract were evaporated to dryness in a round-bottom flask using a boiling water bath. To the dried samples, heated under a reflux condenser (20–30 cm), 10 mL of KOH in methanol (0.2 M) and 1 mL of heptane were added. After 10 min, 5 mL of boron trifluoride (BF_3_) was added and followed, after 2 min, by 4 mL of hexane. After waiting for 1 min, the samples were cooled. A saturated solution of Na_2_SO_4_ was added to the samples, and after settling into a two-phase system, the upper layer was taken and transferred into a vial. The samples were stored at −30 °C until further analysis (Dussault et al. 2009).

GC-MS analysis was performed using a Thermo Trace 1300 Gas Chromatograph equipped with a Thermo TG-WAXMS capillary column (dimensions: 30 m × 0.25 mm × 0.25 μm; Thermo Scientific, USA), coupled to a Thermo ISQ LT Single Quadrupole Mass Spectrometer (MS) through a heated transfer line (220 °C). Helium (99.999%) was used as the carrier gas with a constant flow rate of 1 mL/min and a 1:50 split ratio. The GC inlet temperature is 220 °C. 1 mL aliquots were injected using an AI 1310 autosampler, and the GC oven was programmed to hold 120 °C for 5 min, then raise the temperature by 6 °C/min to 210 °C, which was held for 5 min, then raise the temperature by 1 °C/min to 230 °C, which was held for 20 min. The MS was operated with the ion source at 220 °C. The solvent delay is 1.46 min. FAME peaks were identified by comparison of their retention times with those of a standard solution (GLC NESTLE 37 Component FAME MIX) and quantified with the internal standard. In all cases, the mass spectrometer was operated in the electron ionization mode (EI) at 70 eV. The retention times and the characteristic fragments of the EI mass spectra were obtained from *m/z* 20–400 with the scan rate of 500 amu/s. The most abundant ions and/or ions with-out apparent cross-contribution and interferences were chosen as target ions for the quantification (SIM mode) (Schummer et al. 2009).

### Electrophoretic analysis

All the implements must be precooled at −20 °C before the tests. The membrane protein (MP) was prepared with Bacterial Membrane Extraction Kit (BestBio, Shanghai, China) according to the instructions with minor modification. The working cultures of *S. putrefaciens* with different treatments at 180 min were centrifuged at 10,000*g* for 5 min at 0 °C, then washed with PBS buffer (0.1 M, pH 7.0) for two times. The extraction buffer A (500 μL, combined with 2 μL Protease inhibitor) was added to the sediments (20 mg), then put on ice for 2–3 h with shaking for 30 s with every 30 min. The mixture was then centrifuged at 12,000*g* for 5 min at 0 °C, the supernatant was collected, and then 10 μL. Extraction buffer B was added to the supernatant, then kept at 37 °C for 10 min. The mixture was centrifuged at 1000*g* for 5 min at 37 °C. The under layer samples were the membrane protein (MP). MP concentration was measured with enhanced bicinchoninic acid (BCA) Protein Assay Kit (Beyotime Biotechnology, Jiangsu, China) according to the manufacturer’s instructions. The samples may need to be diluted further to same concentration with appropriate volume of membrane protein dissolution buffer in the kit. The loaded concentration of different treatments’ MP was adjusted to 1 mg/mL. 20 μL of protein lysates was then mixed with 5 μL 5× SDS gel-loading buffer (60 mM Tris–HCl, pH 6.8, 14.4 mM dithiothreitol, 2% SDS, 25% glycerol, and 0.1% bromphenol blue), boiled at 100 °C for 5–10 min before use or stored at −20 °C until use. The loaded volume was 15 μL. Sodium-dodecyl-sulfate polyacrylamide gel electrophoresis (SDS-PAGE) was used to measure the molecular weight changes of MP, using 12% separating gel and a 5% stacking gel. The running buffer was 25 mM Tris-192 mM glycin-0.1% SDS, pH 8.3. The power supply was settled at 80 V during the stacking of the proteins, then at 120 V. Gels were stained with Coomassie Brilliant Blue R250 and destained with a solution of methanol and acetic acid to visualize proteins (Carraro and Catani 1983).

### Changes of cinnamon oil in nutrient broth with *S. putrefaciens*

GC–MS was used to analyze chemical compositions of cinnamon oil in nutrient broth (NB) of C1 at 0 and 180 min. GC–MS analysis was performed using a Thermo Trace GC Ultra system equipped with a Thermo TR-5MS capillary column (dimensions: 30 m × 250 mm × 0.25 mm; Thermo Scientific, Runcorn, UK) operating with helium as a carrier gas, coupled to a Thermo ITQ 1100 mass spectrometer (MS) through a heated transfer line (230 °C). The GC injector (230 °C) was operated in a pulsed split mode (50:1); 1 μL aliquots were injected using an autosampler. The initial oven temperature of GC was 100 °C, and programmed to 130 °C at a rate of 20 °C/min, then programmed to 170 °C at a rate of 5 °C/min, after then, programmed to 230 °C at a rate of 25 °C/min and then kept constant at 230 °C for 5 min. The MS was operated with the ion source at 230 °C and a damping flow of 1 mL/min. MS was taken at 70 eV and a mass range of 35–425 amu. The solvent delay time was 3 min. A library search was carried out using NIST98.L database. Relative percentage amount was calculated from TIC by the computer (Ooi et al. 2006).

### Distribution of cinnamaldehyde and cinnamyl alcohol

After the analysis of cinnamon oil changes in broth in C1, we found that in the nutrient broth, cinnamaldehyde decreased and cinnamyl alcohol emerged (data are shown in results section). Thus, the distribution of cinnamaldehyde and cinnamyl alcohol in nutrient broth and *S. putrefaciens* was analyzed using HPLC system (Waters 2695, Milford, USA) consisting of quaternary gradient pump, autosampler, column oven, and photodiode array detector (PDA, Waters 2996). Chromatographic data were acquired using Empower software. The HPLC column consisted of a Waters symmetry C18 column (200 mm × 4.6 mm × 5 mm) connected to Nova-Pak C18 Guard-PakTM guard column (2 mm × 4 mm × 5 mm). The gradient elution was employed using deionized water and acetonitrile at 30 °C for 20 min. The flow rate was set at 1 mL/min. A volume of 10 μL of sample was injected into HPLC system for analysis. The detection wavelengths were set at 290 and 250 nm for cinnamaldehyde and cinnamyl alcohol, respectively. To calculate their concentration, the standard curves ranging from 0.2 to 200 μg/mL were used to obtain a linear relationship between concentrations of drugs versus peak area response, which resulted in a *R* ^2^ (coefficient of determination) value of 0.9999. A re-equilibration period of 5 min was used between individual runs. Because only treatments of C1, C1+G, and C2 were treated with cinnamon oil, so here the changes of cinnamaldehyde and cinnamyl alcohol of C1, C1+G, and C2 at 0 and 180 min were observed. In order to observe the effect of *S. putrefaciens*, the changes of cinnamaldehyde and cinnamyl alcohol of CC1 and CC2 at 0 and 180 min were also detected.

### Statistical analysis

One-way analysis of variance and Duncan’s multiple ranges tests were employed to determine the effect of the combination of cinnamon oil and GI treatments on fatty acids, cinnamaldehyde, and cinnamyl alcohol. Calculations were performed using SPSS software Base 19.0 (SPSS, Inc., Chicago, IL, USA). Differences between means were considered significant at *p* ≤ 0.05.

## Results and discussion

### GC–MS analysis of cinnamon oil

The chemical compositions of cinnamon oil analyzed by GC–MS method. There were eight primary phytochemicals of cinnamon oil were identified. Trans-cinnamaldehyde was the major compound, according for 78.54%. It has also been reported in previous studies that cinnamaldehyde was the major compound of cinnamon oil (Cheng et al. 2006; Senanayake et al. 1978). Cinnamon oil (*Cinnamomum cassia* Blume) was identified 85% trans-cinnamaldehyde by GC–MS analysis (Ooi et al. 2006). Trans-cinnamaldehyde was also detected in *Cinnamomum osmophloeum* leaves’ constituents, which accounted for 76.00% (Chang et al. 2001).

### Changes of fatty acids in *S. putrefaciens*

**Fig. 1:**
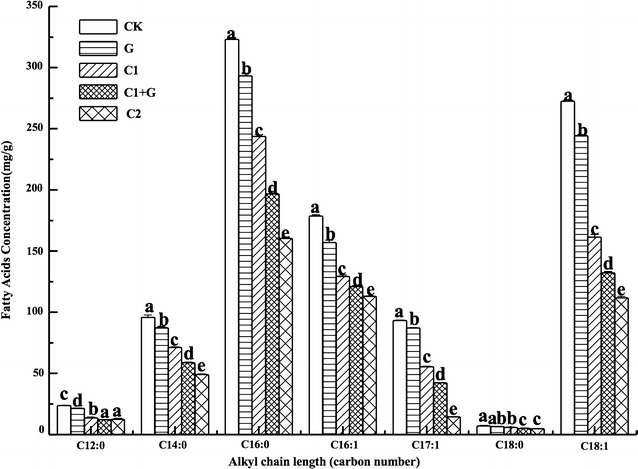
Changes of membrane fatty acids of *S. putrefaciens* with different treatments. CK, *S. putrefaciens* without treatment; C1, *S. putrefaciens* treated with 207 μg/mL cinnamon oil; C2, *S. putrefaciens* treated with 414 μg/mL cinnamon oil; G, *S. putrefaciens* irradiated with 0.080 kGy gamma irradiation dose; C1+G, *S. putrefaciens* treated with 207 μg/mL cinnamon oil and then irradiated with 0.080 kGy gamma irradiation dose

Fatty acid composition of microorganisms at various points is required for understanding membrane-associated processes of cells (Špitsmeister et al. 2010). The changes of membrane fatty acid compositions of *S. putrefaciens* with different treatments at 180 min are shown in Fig. 1. Saturated fatty acids (SFA), C12:0, C14:0, C16:0, C18:0, and unsaturated fatty acids (UFA), C16:1(9c), C17:1(10c), C18:1(9c) were observed in *S. putrefaciens* in our study. Our result is in agreement with Moule and Wilkinson (1987) who reported that *S. putrefaciens* can biosynthesize a variety of fatty acids, such as saturated fatty acids (SFA), C12:0, C14:0, C16:0, C18:0, unsaturated fatty acids (UFA), C16:1(9c), C17:1(10c), C18:1(9c). Wang et al. (2009) reported that *Shewanella* inhabiting various environments contain SFA (16:0, 18:0) and UFA (16:1, 18:1).

Compared with CK, all of FAs of G, C1, C1+G, and C2 showed a decrease trend, especially for C14:0, C16:0, C16:1, C17:1, C18:1 (*p* < 0.05). C2 showed the largest decrease level on these fatty acids, followed by C1+G, C1, and G (*p* < 0.05). The higher concentration of cinnamon oil was, the stronger damage on the membrane of *S. putrefaciens*. The combination of C1+G showed a higher damage on fatty acids than C1 and G alone, and C1 was stronger than G. This indicated that the treatments of cinnamon oil and irradiation had an obvious influence on the membrane of *S. putrefaciens.* Biological membranes are essential for the cell integrity, providing a barrier between the inside and outside environments for the cell (Pedersen et al. 2006), and the membrane lipids play an essential role in microbial adaptation under different environmental changes. Irradiation, cinnamon oil, and the combination treatments all showed obviously effect on the lipid profiles, and the effect of cinnamon oil was stronger than irradiation. This is similar with the study of Di Pasqua et al. (2007) who reported that modifications of the membrane by GI were not important when compared with those treated with antimicrobial agents such as essential oils (i.e., thymol).

The UFA ratio of the untreated *S. putrefaciens* was above 0.50, showed in Table 1. *Shewanella* bacteria naturally reside/inhabit the deep-sea environment with low temperature, the mechanism for their survival lies in that the high percentage of UFA in the bacterial membrane incorporated with phospholipid confers the better membrane fluidity, which in turn enhances its capability of cold adaptation (Zhang et al. 2015a). Di Pasqua et al. (2006) reported that UFAs are always present at a higher amount than SFAs in the total lipid profile of the microorganisms used in their research. Wang et al. (2009) also reported that *Shewanella* has appreciable ability to produce various types of low-melting-point fatty acids with monounsaturated fatty acids (MUFA) included.

**Table 1:** Changes in the concentrations of principal fatty acids of *S. putrefaciens*

Fatty acid indexes	Treatments				
	CK	G	C1	C1+G	C2
UFA ratio	0.548 ± 0.001^a^	0.545 ± 0.002^a^	0.509 ± 0.003^d^	0.520 ± 0.003^b^	0.514 ± 0.003^c^
SFA ratio	0.452 ± 0.001^d^	0.455 ± 0.002^d^	0.491 ± 0.003^a^	0.480 ± 0.003^c^	0.486 ± 0.003^b^
SFA/UFA ratio	0.825 ± 0.008^a^	0.836 ± 0.006^a^	0.966 ± 0.005^d^	0.922 ± 0.012^b^	0.946 ± 0.009^c^

UFA ratio, SFA ratio, and SFA/UFA ratio represent ratio of UFA/TFA (total fatty acids), ratio of SFA/TFA, ratio of SFA to UFA, respectively; CK, *S. putrefaciens* without treatment; C1, *S. putrefaciens* treated with 207 μg/mL cinnamon oil; C2, *S. putrefaciens* treated with 414 μg/mL cinnamon oil; G, *S. putrefaciens* irradiated with 0.080 kGy gamma irradiation dose; C1+G, *S. putrefaciens* treated with 207 μg/mL cinnamon oil and then irradiated with 0.080 kGy gamma irradiation dose; Values are the mean ± standard deviation of the weight percentages of fatty acids in total lipid isolated from at least three independent cultures. Different letters mean significantly different (*p* < 0.05) data in the same row

The saturated/unsaturated fatty acids (SFA/UFA) ratio can effectively demonstrate the destructive effect of different treatments on the membrane fatty acids. The SFA/UFA ratios are shown in Table 1. The SFA/UFA ratios of CK, G, C1, C1+G, and C2 were 0.825, 0.836, 0.966, 0.922, and 0.946, respectively. CK and G showed a lower SFA/UFA ratio values than C1, C1+G, and C2 (*p* < 0.05). The increase of SFA/UFA ratio indicated a reduction of UFA or an increase of SFA. Significant decrease of UFA ratio and increase of SFA ratio took place in C1, C1+G, and C2 treatment, as compared with CK and G (*p* < 0.05). This result revealed that the antimicrobial mechanism of cinnamon oil against the cell membrane of *S. putrefaciens*, interacting with the membrane lipid profile and causing membrane structural alterations. It is well known that UFAs give the membrane a high degree of fluidity, thus affecting the adaptive capacity of bacteria. The decrease of UFA and increase of SFA were detected in *Pseudomonas fluorescens* and *Staphylococcus aureus* treated with a sublethal concentration of some essential oil antimicrobial compounds (Di Pasqua et al. 2006).

### Electrophoresis of the membrane protein (MP) of *S. putrefaciens*

**Fig. 2:**
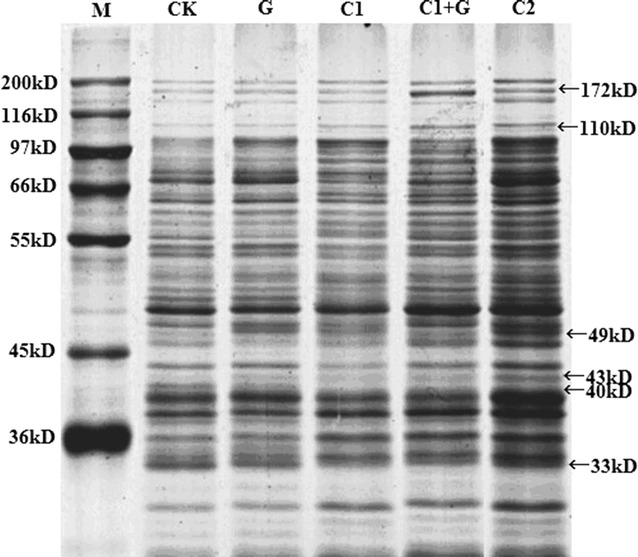
SDS-PAGE fractionation of *S. putrefaciens* membrane proteins with different treatments. The sampling amounts of the SDS-PAGE for each lane contained equal protein content based on BCA Protein Assay Kit (Beyotime). M, Marker; CK, *S. putrefaciens* without treatment; C1, *S. putrefaciens* treated with 207 μg/mL cinnamon oil; C2, *S. putrefaciens* treated with 414 μg/mL cinnamon oil; G, *S. putrefaciens* irradiated with 0.080 kGy gamma irradiation dose; C1+G, *S. putrefaciens* treated with 207 μg/mL cinnamon oil and then irradiated with 0.080 kGy gamma irradiation dose. Molecular masses were estimated from commercial standards (Sigma Chemical Co.)

SDS-PAGE analysis was performed to investigate the effect of cinnamon oil and GI on protein changes of *S. putrefaciens* (Katayama et al. 2002). Figure 2 shows the changes in the MP subunits during different treatments with the same sampling protein content and same volume. The loading concentration of proteins was adjusted according BAC Protein Assay Kit (see method section of electrophoretic analysis). More than 30 protein bands in the MP of *S. putrefaciens* were resolved ranging in size from 30 to 172 kD as determined by visual assessment of their approximate molecular masses. MP of C1, C1+G, and C2 showed more obvious brands than CK and G, especially the higher concentration cinnamon oil. It indicated that the secondary or tertiary structure of membrane proteins of C1, C1+G, and C2 were more affected with more exposure of amino and sulfhydryl groups in membrane proteins, and thus more Coomassie dye R-250 was bound to protein or protein subunit (Knauf and Rothstein 1971).

### Changes of cinnamon oil in nutrient broth with *S. putrefaciens*

**Fig. 3:**
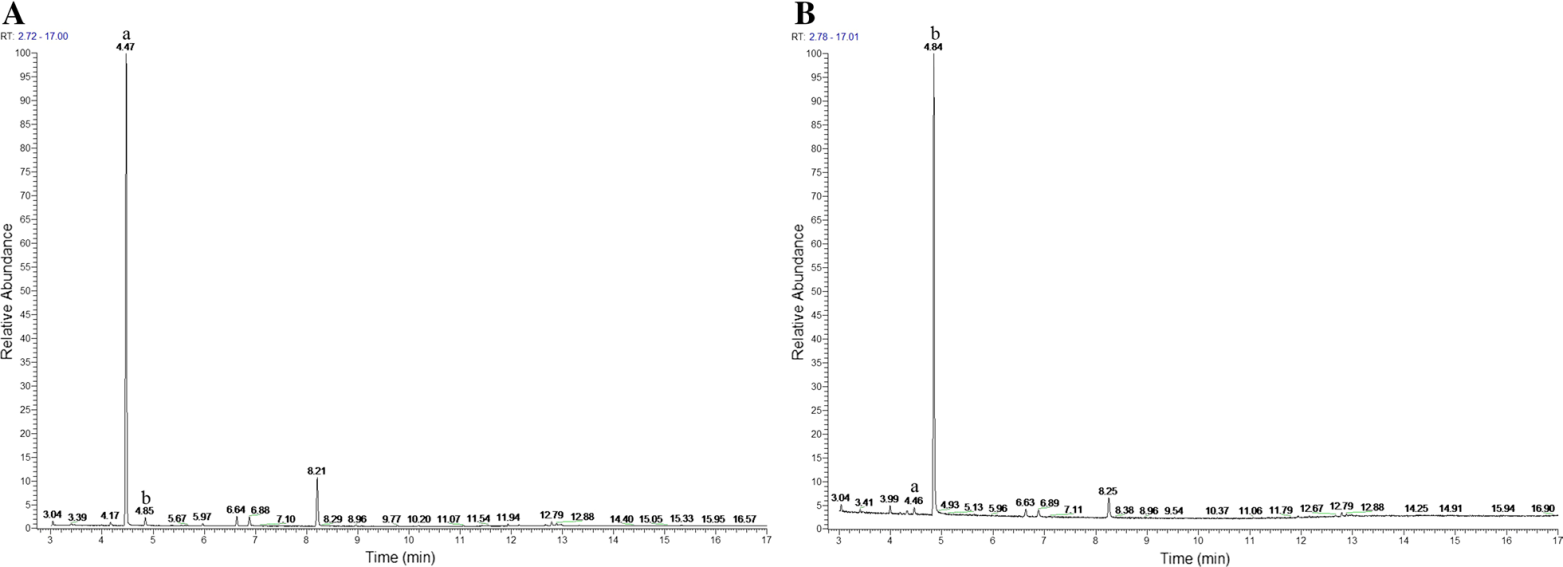
Changes of chemical compositions of cinnamon oil in nutrient broth of treatment C1 by GC–MS analysis at 0 (**A**) and 180 min (**B**). *a* Trans-cinnamaldehyde, *b* cinnamyl alcohol. C1, *S. putrefaciens* treated with 207 μg/mL cinnamon oil

Figure 3 shows component changes of cinnamon oil in nutrient broths at 0 and 180 min. At 0 min, the main component in the nutrient broth was cinnamaldehyde (peak a in Fig. 3A), accounting for 82.41%. After 180 min, cinnamaldehyde reduced obviously to 1.93% (peak a in Fig. 3B), while the main component in the nutrient broth changed into cinnamyl alcohol (peak b in Fig. 3B), which increased from 2.03% at 0 min to 82.04% at 180 min (peak b in Fig. 3). Cinnamyl alcohol can be prepared by selective hydrogenation of cinnamaldehyde though biocatalysis of enzymes and microorganisms (Hollmann et al. 2011). *Bacillus stearothermophilus* alcohol dehydrogenase can reduce cinnamaldehyde to cinnamyl alcohol with the consumption of reduced form of nicotinamide-adenine dinucleotid (NADH) (Pennacchio et al. 2013). The transformation of cinnamaldehyde to cinnamyl alcohol in *S. putrefaciens* nutrient broth would support the theory of selective hydrogenation of cinnamaldehyde catalyzed by enzymes or microorganisms.

### Distributions of cinnamaldehyde and cinnamyl alcohol in nutrient broth and *S. putrefaciens*

**Fig. 4:**
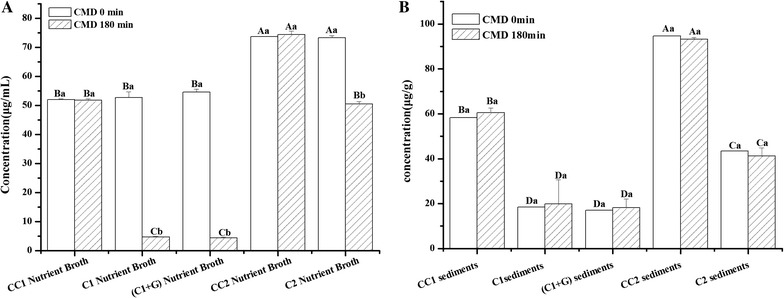
Changes in cinnamaldehyde concentrations in nutrient broth (**A**) and *S. putrefaciens* (**B**) with different treatments. CC1, control with 207 μg/mL cinnamon oil but without *S. putrefaciens*; CC2, control with 414 μg/mL cinnamon oil but without *S. putrefaciens*; C1, *S. putrefaciens* treated with 207 μg/mL cinnamon oil; C2, *S. putrefaciens* treated with 414 μg/mL cinnamon oil; C1+G, *S. putrefaciens* treated with 207 μg/mL cinnamon oil and then irradiated with 0.080 kGy gamma irradiation dose. *Uppercase letters* the significant difference (*p* < 0.05) of different treatments within the same time; *lowercase letters* the significant difference (*p* < 0.05) of the same treatments within different times

The distributions of cinnamaldehyde and cinnamyl alcohol of all cinnamon oil treatments in nutrient broth and *S. putrefaciens* are shown in Fig. 4. As shown in Fig. 4A, after 180-min treatment of cinnamon oil, the cinnamaldehyde concentrations of C1, C1+G, and C2 in the nutrient broth decreased obviously as compared with them at 0 min. As concerned on the controls of CC1 and CC2 contained 207 and 414 μg/mL cinnamon oil without *S. putrefaciens*, almost no change of cinnamaldehyde concentration was observed in nutrient broth from 0 to 180 min. This result proved that cinnamaldehyde was transformed to cinnamyl alcohol with *S. putrefaciens*.

Distribution of cinnamaldehyde in *S. putrefaciens* was shown in Fig. 4B. Cinnamaldehyde was detected in the sediments of CC1 and CC2 at 0 and 180 min, indicating that the sediments without bacteria cells contain cinnamon oil. While cinnamaldehyde concentrations of C1, C1+G, and C2 treatments containing bacterial cells were lower than CC1 and CC2, respectively, no matter at 0 or 180 min. There were no significant differences in the treatments of C1, C1+G, and C2 both at 0 and 180 min. It may be due to that cinnamon oil cannot pass through the bacterial membrane, or even little of them pass the membrane; it may be metabolized into the other components as soon as possible.

**Fig. 5:**
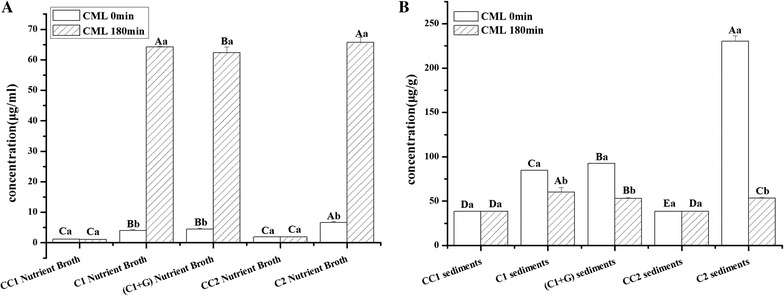
Changes in cinnamyl alcohol concentrations in nutrient broth (**A**) and *S. putrefaciens* (**B**) with different treatments. CC1, control with 207 μg/mL cinnamon oil but without *S. putrefaciens*; CC2, control with 414 μg/mL cinnamon oil but without *S. putrefaciens*; C1, *S. putrefaciens* treated with 207 μg/mL cinnamon oil; C2, *S. putrefaciens* treated with 414 μg/mL cinnamon oil; C1+G, *S. putrefaciens* treated with 207 μg/mL cinnamon oil and then irradiated with 0.080 kGy gamma irradiation dose. *Uppercase letters* the significant difference (*p* < 0.05) of different treatments within the same time; *lowercase letters* the significant difference (*p* < 0.05) of the same treatments within different times

Changes of cinnamyl alcohol of all cinnamon oil treatments in broth and in *S. putrefaciens* are shown in Fig. 5. The cinnamyl alcohol concentration of treatments of C1, C1+G, and C2 in nutrient broth increased at 0 or 180 min as compared with CC1 and CC2, especially at 180 min. As concerned for the cinnamyl alcohol concentration at 180 min, there were obviously increases in C1, C1+G, and C2 in nutrient broth (Fig. 5A). This result was consistent with the changes of cinnamaldehyde as shown in Fig. 4A. It is the first time to report that cinnamaldehyde was transformed into cinnamyl alcohol in the presence of *S. putrefaciens*. The changes of cinnamyl alcohol concentration in *S. putrefaciens* of different treatments are shown in Fig. 5B. The cinnamyl alcohol concentrations of C1, C1+G, and C2 at 0 min were obviously higher than that of CC1 and CC2, respectively. It means that with the action of *S. putrefaciens*, the cinnamaldehyde in the cell can be quickly transformed into cinnamyl alcohol. But at 180 min, cinnamyl alcohol concentrations of C1, C1+G, and C2 decreased obviously.

Because of the hydrophobic character of cinnamon oil, it was not easy to transfer the membrane of *S. putrefaciens* for cinnamaldehyde. The changes of cinnamaldehyde and cinnamyl alcohol in broth and *S. putrefaciens* indicated that the biotransformation of cinnamaldehyde to cinnamyl alcohol occurred on the membrane of *S. putrefaciens*. With transformation of cinnamaldehyde to cinnamyl alcohol, obvious damages of fatty acids of *S. putrefaciens* were also found in our study. All these illustrated that the action site of cinnamon oil appears to be on the membrane of *S. putrefaciens.* GI could help cinnamon oil to damage the membrane lipid and protein profile, but no effect on the transformation of cinnamaldehyde.

## Conclusion

In order to detect the antimicrobial mechanism of the combination of cinnamon oil and GI on *S. putrefaciens*, the changes of cinnamaldehyde, membrane fatty acids, and proteins of *S. putrefaciens* were observed. The changes of membrane fatty acids and proteins of treated with C1+G were obvious than CK, C1, and G. Cinnamon oil or the combination with GI could affect the membrane proteins. Interestingly, after cinnamon oil treatments, the content of cinnamaldehyde in the nutrient broth decreased, and cinnamyl alcohol increased obviously. The antimicrobial mechanism might be complex, with the action of GI, the destruction ability of cinnamon oil on membrane fatty acids and proteins increased. Cinnamaldehyde was transformed into cinnamyl alcohol with *S. putrefaciens*. It might be catalyzed by alcohol dehydrogenase with consumption of NADH in *S. putrefaciens*, thus the activity of *S. putrefaciens* also might be inhibited with the transformation of cinnamaldehyde.

## Abbreviations

*S. putrefaciens*: *Shewanella putrefaciens*
GI: gamma irradiation
MP: membrane proteins
SDS-PAGE: sodium dodecyl sulfate polyacrylamide gel electrophoresis
SSO: specific spoilage organisms
H_2_S: hydrogen sulfide
TMAO: trimethylamine oxide
TMA: trimethylamine
EO: essential oil
GRAS: generally recognized as safe
ATP: adenosine triphosphate
DNA: deoxyribonucleic acid
ROS: reactive oxygen species
GCP: chromatographic pure
NB: nutrient broth
NA: nutrient agar
CFU: colony-forming unit
CK: control, *S. putrefaciens* without any treatments
C1: treatment, *S. putrefaciens* were added 207 μg/mL cinnamon oil
C1+G: treatment, *S. putrefaciens* were added 207 μg/mL cinnamon oil and then and irradiated with 0.080 kGy
G: treatment, *S. putrefaciens* were gamma irradiated with 0.080 kGy
C2: treatment, *S. putrefaciens* were added 414 μg/mL cinnamon oil
MIC: minimal inhibitory concentration
GC–MS: gas chromatography-mass spectrometer
CC1: control, nutrient broths containing 207 μg/mL cinnamon oil without *S. putrefaciens*
CC2: control, nutrient broths containing 414 μg/mL cinnamon oil without *S. putrefaciens*
HPLC: high performance liquid chromatography
TIC: total ions chromatograms
FAME: fatty acid methyl esters
GC: gas chromatography
MS: mass spectrometer
BCA: bicinchoninic acid
PDA: photodiode array detector
SFA: saturated fatty acids
UFA: unsaturated fatty acids
TFA: total fatty acids
MUFA: monounsaturated fatty acids
NADH: reduced form of nicotinamide-adenine dinucleotide
